# A theoretical review of interpersonal emotion regulation in eating disorders: enhancing knowledge by bridging interpersonal and affective dysfunction

**DOI:** 10.1186/s40337-020-00298-0

**Published:** 2020-06-01

**Authors:** Kara A. Christensen, Ann F. Haynos

**Affiliations:** 1 Department of Psychology, University of Kansas, 1415 Jayhawk Blvd Rm 440, Lawrence, KS 66045 USA; 2 Department of Psychiatry and Behavioral Sciences, University of Minnesota, 2450 Riverside Ave, F253, Minneapolis, MN 55454 USA

**Keywords:** Interpersonal relationships, Emotion regulation, Interpersonal emotion regulation, Eating disorders

## Abstract

Individuals with eating disorders (EDs) frequently report interpersonal and affective dysfunction. A useful lens for uniting these ideas is through the framework of interpersonal emotion regulation (IER), which consists of the ways others assist a distressed individual and how this shapes his or her subsequent emotional, behavioral, and cognitive responses. In this theoretical review, we provide an overview of the rationale for exploring IER and review IER processes in this population using the framework of the Process Model of Emotion Regulation. Finally, we offer suggestions for next steps in conducting research. IER offers a parsimonious way to explore social and emotional constructs related to ED pathology and may provide potential targets for prevention and intervention in these difficult-to-treat disorders.

## Plain English summary

People with eating disorders frequently report difficulties in their relationships and experiencing difficult-to-control emotions. We propose that studying the different ways that people receive help with managing their emotions (i.e., interpersonal emotion regulation) is promising for better characterizing the specific problems that people with eating disorders experience. In this review paper, we discuss the current research on interpersonal emotion regulation and future directions for this field. Increased knowledge of interpersonal emotion regulation could lead to the development and/or refinement of prevention and intervention methods that specifically target maladaptive social support behaviors linked to emotional distress.

## Introduction

**Fig. 1:**
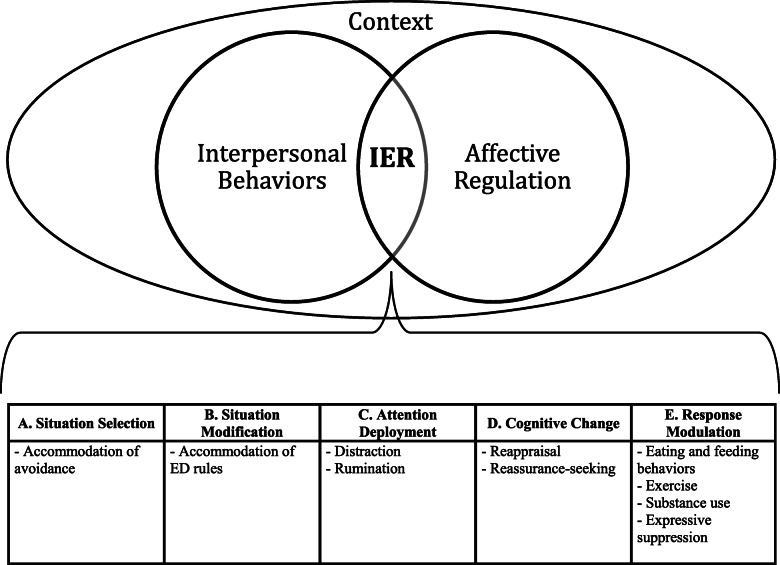
Process Model of Interpersonal Emotion Regulation in Eating Disorders. Interpersonal emotion regulation (IER) is at the intersection of interpersonal behaviors and affective regulation. To evaluate if interpersonal behaviors, affective regulation, and interpersonal emotion regulation are adaptive or maladaptive, one must consider contextual factors such as personal goals, culture, demographics, and stage of recovery

Individuals with eating disorders (EDs) report both interpersonal and affective dysfunction [1–3], which may be united by a growing literature exploring a construct called interpersonal emotion regulation (IER; Fig. 1). IER consists of the ways in which people intentionally engage with an individual to modify the individual’s emotions and how this shapes subsequent emotional, behavioral, and cognitive responses of each member of the dyad [4–9]. Although there is a long history of interest in how different aspects of close interpersonal relationships, such as feelings of security or warmth, influence the emotional health of others (e.g., attachment theory [10, 11], interpersonal theory [12], object-relations theory [13], cognitive-interpersonal model of anorexia nervosa [14, 15]), there has been considerably less work evaluating the specific strategies that people use interpersonally to modify emotional experiences.

IER is a broad framework for understanding interpersonal emotion management dynamics that can be applied in both non-clinical and clinical contexts and is complementary to existing models of ED etiology and maintenance. Of note, IER shares similarities with other related constructs, such as attachment, social support, and emotional contagion, but differs in that it requires intentionality in trying to modify emotional response by the regulator [4]. For instance, if a person is nervous about a presentation, she may turn to a friend for reassurance, which thereby reduces her anxiety. In turn, the friend’s distress caused by their friend’s anxiety may also be reduced, resulting in a bidirectional, interactional process between the two members of the dyad. These types of interactions are common to many relationships and frequently benefit both members of the dyad; however, when IER is used inflexibly, inefficiently, or inappropriately, it may result in dysfunction for the individuals and their relationship. In this way, IER may serve as a risk and maintenance factor for psychopathology.

Indeed, the IER framework has been applied to explain the etiology and maintenance of psychological problems such as depression [8, 9] and anxiety disorders [8]. It has significantly expanded the understanding of potential prevention and treatment targets; however, it has yet to be applied to EDs. Studying IER may provide additional clarity in how problems in emotion regulation (ER) processes interact with interpersonal problems to promote ED behaviors. Information about IER adds explanatory value above and beyond either interpersonal or emotional problems by better capturing the dynamic ways in which people interact with each other for emotional purposes. Furthermore, information about IER may help to improve the specificity of existing models of ED etiology and maintenance by providing a framework for understanding problematic interpersonal and emotional behaviors.

In this review, we will provide evidence supporting the utility of studying IER in EDs based on interpersonal and affective dysfunction risk factors, describe the status of this area of study, and offer directions for future research. To do this, we will describe evidence for global use of IER strategies (e.g., co-rumination about general anxieties within social relationships) by individuals with EDs, as well as disorder-specific manifestations of IER (e.g., interpersonal facilitation of food avoidance to reduce anxiety).

### Interpersonal problems in EDs

Individuals with EDs frequently report difficulties with social functioning [1].^1^ These interpersonal difficulties may serve as both risk or maintenance factors for eating pathology, or could simply constitute interpersonal consequences of ED behavior. In terms of risk factors, at the trait level, people with EDs endorse more maladaptive interpersonal personality profiles, such as overly nurturing and accommodating, nonassertive, dependent, and socially avoidant styles [16]. People with EDs report less pleasure from social encounters and a lower interest in social interaction [17]. In prospective studies, low social support has been found to confer risk for future binge eating [18], and to interact with negative life events to predict increased bulimic symptoms [19]. Alternately, greater social support serves as a protective factor to mitigate the effects of ED risk factors [20]. ED behaviors also may produce strains on relationships that result in stressful interactions or fractures and may maintain illness. In a study of undergraduate roommate pairs, the roommates of women who endorsed higher bulimic symptoms reported lower intentions to continue living together [21]. Furthermore, carers of those with EDs tend to report high levels of psychological distress and burden [22]. Additional interpersonal problems that may be associated with EDs and affective dysfunction include, but are not limited to, social withdrawal [23, 24], perfectionism [25, 26], and interpersonal distrust [27–29].

There appears to be a bidirectional effect between interpersonal problems and ED behaviors, wherein the two may exacerbate each other, resulting in increased interpersonal dysfunction for the individual and a worsening of psychological symptoms [30, 31]. Higher levels of interpersonal problems at the onset of therapy have been associated with poorer recovery rates [32], underscoring the need to address these difficulties.

### Emotion regulation difficulties in EDs

Research suggests that difficulties in emotion regulation (i.e., managing the onset, intensity, and duration of emotions) influence risk and maintenance of EDs [33, 34]. The habitual use of specific ER strategies, such as avoidance, rumination, and expressive suppression has been linked to increased ED severity, whereas the habitual use of reappraisal, problem-solving, and acceptance are associated with lower symptoms [35]. Importantly, emotional dysregulation in EDs may be both a risk and maintenance factor for eating pathology. For instance, emotion dysregulation in EDs may represent a global risk factor for psychopathology, a disorder-specific risk factor, or a combination of the two. In fact, some hypothesize that ED behaviors are secondary ER strategies that occur to manage negative affect states due to the lack of access to effective ER strategies and/or an overreliance on ineffective strategies [2, 3]. More research is needed to evaluate if emotion dysregulation in EDs is best characterized by global problems in managing affect, inappropriate ways of managing emotions (such as ED behaviors) generated by eating, shape, weight, or interpersonal stimuli, or an interaction between the two.

The literature on ER strategy usage in EDs has primarily focused on it from an *intra*personal perspective, examining how individuals with EDs manage their emotional experiences on their own (e.g., suppressing one’s emotions when viewing a television program) and in *inter*personal contexts (e.g., suppressing one’s facial expressions in front of a romantic partner). This approach is limited, as people frequently turn to others for assistance in managing their distress [4, 6, 7, 36, 37]. Although previous reviews have examined *intra*personal ER difficulties in EDs [2, 3, 38] and IER in healthy individuals [4, 37], there has yet to be a review that synthesizes the specific strategies that are used in interpersonally to effectively or ineffectively regulate emotions in people with EDs. Given the interpersonal and affective dysfunction associated with EDs, it is critical to construct and evaluate a comprehensive theory of IER (Fig. 1).

### Process model of IER in ED populations

In this paper, we use the Process Model of Emotion Regulation [39] to describe IER in EDs. Although there are other frameworks that characterize emotion dysregulation [40], we chose the Process Model due to its large body of supporting research and use of specific strategies rather than domains of dysfunction (e.g., “lack of access to strategies”), permitting more nuanced examination of how these strategies may be used interpersonally.

**Fig. 2:**
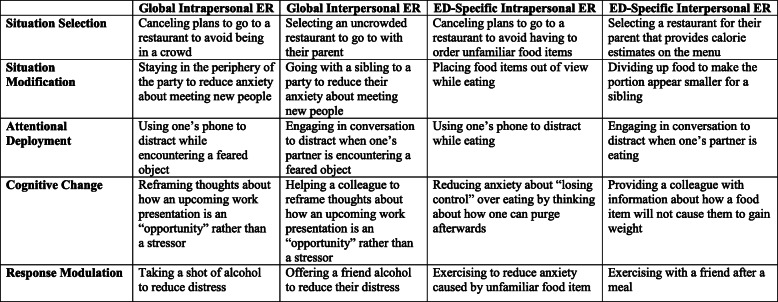
Extending the Process Model to conceptualize intrapersonal and interpersonal emotion regulation in EDs. Both intrapersonal and interpersonal ER can exist in global or ED-specific contexts

According to the Process Model, ER strategies can be divided into: 1. Situation selection; 2. Situation modification; 3. Attentional deployment; 4. Cognitive change; and 5. Response modulation. Although these categories have primarily been examined as *intra*personal constructs, they can also be *inter*personal (Fig. 2). Further, as we have noted, these emotion regulation processes could directly involve ED-specific content and contexts, or could be more global in nature.

Given the wide range of potential IER behaviors, this review should not be considered exhaustive, but rather, a starting point for exploring this construct. Furthermore, strategies should not be assumed a priori to be adaptive or maladaptive; instead, context, such as personal goals, culture, demographics, and stage of recovery, guides if outcomes are adaptive (Fig. 1) [41]. We approach our model using a transdiagnostic framework, such that these processes may occur across both ED and non-ED diagnostic groups. IER has been theorized to explain emotional and interpersonal problems related to both depression [8, 9] and anxiety disorders [8], although it has yet to be applied to EDs. We believe that the IER framework, like that of intrapersonal emotion regulation describes processes that apply across all types of psychopathology; however, more research is necessary to determine if distinct strategies differentiate specific ED presentations, or ED presentations from other psychiatric concerns.

### Situation selection

Situation selection refers to the ways in which individuals limit exposure to distress-eliciting stimuli. Viewed through an IER lens, situation selection could involve helping an individual avoid exposure to or removing someone from a situation that elicits an emotional response (Fig. 1a). The research on interpersonal facilitation of avoidance has primarily drawn from the literature on accommodation, which has been studied extensively in obsessive-compulsive disorder [42]. Through accommodation, the support system may maintain the illness by actively or passively allowing the person with an ED to engage in behaviors that do not promote recovery (e.g., avoiding challenging foods). In a systematic review, researchers found that accommodation of ED behaviors by caregivers was positively associated with duration of illness, suggesting the establishment of interpersonally-facilitated avoidance patterns over time [43]. Furthermore, in a study of parents of children with anorexia nervosa, accommodation had a dose-dependent effect, with poorest outcomes observed in children with two accommodating parents [44]. Overall, the research on accommodation suggests that avoidance may be an IER strategy adopted among carers of individuals with EDs that maintains illness. Other maladaptive interpersonal situation selection strategies, such as removing individuals from distress-eliciting situations, both global (e.g., school or work) and disorder-specific (e.g., going to a restaurant), have not yet been extensively investigated in EDs and warrant further research.

### Situation modification

Situation modification consists of altering characteristics of an existing context in order to lower emotional response (Fig. 1b). For example, in a non-ED context, a support member could go with the person with an ED to a party, to reduce the anxiety brought up by a social situation. In a disorder-specific context, following ED rules can also be conceptualized as situation modification. Using situation modification as an IER strategy could consist of encouraging “safety” behaviors that maintain the ED (e.g., serving smaller portion sizes).

Evidence suggest that when these behaviors are undertaken, the motivation is not typically to promote the ED, but rather fear of an alliance rupture with the ill individual or to prevent more harmful behavior, such as purging or meal skipping [22].

### Attentional deployment

Attentional deployment reflects how people direct attentional resources to modify the emotional impact of stimuli (Fig. 1c). Both distraction and rumination are considered to be forms of attentional deployment. The interpersonal use of distraction comprises of helping an individual to direct attention away from the stimuli towards other information, whereas interpersonal rumination is a cognitive process in which one directs the individual to focus on the causes or consequences of thoughts, feelings, or memories.

#### Distraction

There is little research that has examined the impact of other individuals providing distraction to those with EDs when they are experiencing negative affect; however, treatments such as dialectical-behavior therapy [45] and family-based therapy [46] suggest using this strategy to promote effective behavior (e.g., avoiding purging). Often distraction is employed in the context of the therapeutic meal; however, there is little research informing optimal strategies for meal support [47]. It is unclear whether distraction is a feasible long-term strategy or may slow food-related habituation. The employment of distraction as an IER strategy may require thoughtful consideration of the goals of usage (e.g., long-term reduction of fear vs. facilitating nutritional rehabilitation). It is probable that the utility of interpersonal distraction varies significantly by the context, such as weight status and stage of recovery. Distraction may also be used for affect that is not specifically related to the ED, such as distraction from other unpleasant emotions, such as anger at a colleague or anxiety related to a test.

#### Rumination

Individuals frequently engage in rumination in an attempt to exert control over emotional distress [48], although it tends to maintain negative affect [49]. Interpersonal use of rumination, also referred to as co-rumination may take two forms: mutual brooding (i.e., passively dwelling on the causes and consequences of an action) and mutual reflection (i.e., actively analyzing in order to problem-solve). Further, the content may take the form of general co-rumination (e.g., focusing on a personal problem) or ED-relevant co-rumination (e.g., engaging in “fat talk”).

The general form of co-rumination has been linked to higher internalizing symptoms [50]. Although co-rumination has been associated with greater psychological difficulty concurrently and prospectively, there are no current studies examining general co-ruminative practices in individuals with EDs. Instead, the ED field has primarily focused on ED-relevant body-related co-rumination such as “fat talk”, in which people interactively share and dwell on negative thoughts and feelings about their bodies [51]. Fat talk has been associated with a variety of negative consequences [52], including higher body dissatisfaction [53], negative affect [54, 55], depression [56, 57], and body checking [42], and lower self-esteem [57]. Fat talk can also be “contagious”; when people are exposed to someone engaging in fat talk, they are more likely to make disparaging comments about their own bodies and report higher negative affect and ED symptoms [43, 46, 47]. Taken together, research points to body-related co-rumination as a problematic interpersonal behavior that may amplify psychological symptoms, which makes it a promising target for intervention.

### Cognitive change

Cognitive change strategies are those that are used to alter how individuals think about a situation, thereby changing its emotional impact (Fig. 1d). Interpersonal use of reappraisal and reassurance-seeking are two IER strategies that may be relevant to ED populations.

#### Reappraisal

Interpersonal reappraisal is a strategy whereby an individual helps another to change their cognitive interpretation of a situation to modulate affective response. Interpersonal reappraisal has been associated with better outcomes to acute stressors in a laboratory study [58]. Similarly, in young children, greater mother-child reappraisal was associated with better affective outcomes [59]. However, there is little other research examining reappraisal interpersonally; rather, previous studies have examined the broader construct of positive interpersonal behavior [60], which comprises a variety of domains including reappraisal, supportive listening, and validation.

There is no published research examining the use of reappraisal as a dyadic process in individuals with EDs. This is a significant knowledge gap, given that intrapersonal reappraisal is foundational to cognitive-behavioral approaches and therapists frequently employ thought challenging with their clients in therapy [61]. Understanding how supports or therapists can more effectively utilize reappraisal with the person with the ED when they are experiencing distress has great potential in improving treatment approaches.

#### Reassurance-seeking

Reassurance-seeking involves an individual repeatedly requesting information from others about a perceived threat in the environment [62]. Reassurance-seeking functions through negative reinforcement such that reassurance from others decreases anxiety and increases the likelihood of future reassurance-seeking. This behavior becomes problematic when the need for reassurance does not end when the feedback is provided; instead many individuals doubt the quality or genuineness of the provided reassurance and seek more reassurance, resulting in interpersonal strain and decreased quality of future feedback [63].

Reassurance-seeking has numerous negative emotional and interpersonal consequences. In one longitudinal study of female college students, reassurance-seeking predicted ED symptoms [64]. In another study of undergraduate students, reassurance-seeking mediated the relationship between bulimic symptoms and longitudinal interpersonal stress [21]. Finally, in a third study, those higher in reassurance seeking had a stronger positive relationship between social avoidance and ED psychopathology [57]. Of note, in these studies, reassurance-seeking was assessed by way of general tendencies to seek reassurance, rather than reassurance specifically related to ED concerns such as shape or weight. The negative interpersonal consequences of excessive reassurance-seeking may be particularly problematic for individuals with EDs, who report reduced social support [65–68].

### Response modulation

Response modulation consists of strategies to alter the quality or intensity of an emotion after it has been initiated. Although there are many forms of response modulation, we have selected a couple strategies that may be of particular relevance to EDs (Fig. 1e).

#### Expressive suppression

Expressive suppression consists of masking facial displays of emotion. Much of the experimental and self-report research on expressive suppression has examined its use in non-social contexts (e.g., individuals suppressing facial expressions to emotion-eliciting videos). This does not capture the instances in which expressive suppression is undertaken to address perceived social concerns about the appropriateness of displaying emotions. Individuals with higher levels of ED symptoms endorse greater beliefs that emotional expression is undesirable and will lead to social rejection [69] and, consequently, are more likely to avoid expressing emotions [70]. Although it is likely that these messages have been reinforced in prior interpersonal relationships, to our knowledge there is no literature specifically examining expressive suppression as an IER strategy in EDs.

#### Eating disorder behaviors

Exploring how others may facilitate problematic eating patterns to regulate emotions is critical for understanding the development and maintenance of EDs. This type of behavior may begin in childhood and have consequences into adolescence and adulthood. Emotional feeding, or hedonic feeding by a caregiver to soothe negative emotions, has been associated with greater emotional eating in children [71, 72] and adolescents [73] and may continue into adulthood [74, 75]. Given that emotional eating is a risk factor for binge eating [18, 76], this IER strategy is of concern.

Although less frequently studied, exercise may also be utilized for ER to reduce negative affect and increase positive affect [77]. There is no research directly examining the link between encouraging exercise for others as an ER strategy and ED outcomes. Instead, the literature primarily focuses on facilitating exercise as a health behavior promoting physical wellness [78]; however, this represents an area warranting further research.

## Discussion

There is a solid foundation to support the utility of further investigating IER in EDs; however, there are many areas where the knowledge base could be expanded. One of the primary questions that exists regarding IER in EDs is whether IER problems represent a global vulnerability to psychopathology, a disorder-specific risk factor, or an interaction between the two. Furthermore, it is unknown how IER may be shaped by the experience of EDs; for instance, if certain IER patterns develop as a result of experiencing the disorder (e.g., people engaging in fat talk then begin using more reassurance-seeking in non-ED domains). Overall, better characterizing how IER contributes to the development and maintenance of EDs could lead to improvements in our theoretical models and psychological treatments. Below we outline several research areas that would enhance the knowledge of IER processes in EDs.

### Definition

Refining the definitions of IER processes is essential to providing a solid foundation for this research. Although the Process Model provides a starting place for conceptualizing IER processes, it was created as a framework for *intra*personal, not *inter*personal ER. The degree to which intrapersonal and interpersonal ER strategies align remains unknown. Therefore, more research is needed to identify the various ways in which social environments contribute to the regulation of emotions. An initial step involves qualitative and quantitative studies of the different ways that people with EDs and their social supports engage in IER. In this line of research, it would be important to differentiate strategies using to regulate general vs. ED-specific distress. It is possible that ED-specific forms of IER may exert differential (perhaps stronger) effects on affect and well-being for individuals with EDs. Alternately, ED-related IER may simply reflect broader underlying dysfunction. Future research is needed to delineate general and ED-specific IER strategies, how they interact, and how each influence ED symptoms.

### Assessment

The next step in IER research involves refining assessment measures and techniques. There are existing questionnaires to examine general patterns of IER, such as the Interpersonal Emotion Regulation Questionnaire by Williams and colleagues [5] and the Interpersonal Emotion Regulation Questionnaire by Hofmann and colleagues [79], which provide information about the frequency and utility of IER, but do not provide information about the use of specific strategies. Other measures that capture IER constructs, such as the Accommodation and Enabling Scale for Eating Disorders [80], were not created for the evaluation of IER and therefore, their psychometric properties have not been assessed with these constructs in mind. Thus, there is a need to refine or develop questionnaire measures for assessing general and ED-related IER.

There also is a need to assess IER through naturalistic and experimental designs in order to circumvent memory biases inherent in self-report. Ecological momentary assessment could allow examination of IER strategies in real-time, reducing retrospective bias and permitting investigations of temporal relations between IER, and affective and behavioral outcomes. Observational studies examining how dyads interact when the individual with the ED is upset could also determine the interaction patterns associated with improved or worsening affective and behavioral responses. Examining both short- and long-term outcomes is critical, as changes in short-term affect or behavior may not map onto long-term outcomes. For instance, it may be useful to examine whether certain IER strategies (e.g., accommodating an individual’s ED rule at meals) may reduce short-term negative affect, but ultimately maintain symptoms.

### Context

Context is another important consideration when evaluating IER. There are a variety of factors that may influence the adaptiveness of deploying certain ER strategies [41, 81, 82], including demographics (e.g., race, gender), goals, and setting. Although meta-analyses have found that the habitual intrapersonal uses of certain strategies have positive or negative associations with psychopathology [35], not every “adaptive” strategy is appropriate in every situation and similarly, not every “maladaptive” strategy will have negative consequences [83]. Consequently, it is overly simplistic to categorize strategies as strictly adaptive or maladaptive and a thorough review of contexts and desired outcomes is necessary.

Demographic factors and culture are important contextual variables to consider when evaluating adaptation. For instance, there may be differences in the effectiveness of IER strategies when the person with an ED is a child compared to an adult and depending on the type of relationship between the dyad (e.g., parent-child, romantic partners, colleagues, acquaintances). Individual differences related to the person providing regulation may also drive the success of IER. For instance, it is possible that individuals high in expressed emotion (i.e., high in criticism, low in warmth, high in overinvolvement) may provide less effective or helpful IER to others; similarly, those low in empathy or theory of mind may be limited in their ability to recognize needs for IER and successfully execute these interpersonal behaviors. Similarly, these characteristics may alter the probability that the person with the ED solicits IER from certain individuals.

The appropriateness of IER may also vary across ED subtype and recovery status (e.g., actively ill versus remitted). For example, it is possible that IER strategies of facilitating distraction and avoidance (e.g., of feared foods) may be appropriate during nutritional rehabilitation, but function as safety behaviors once someone is in weight maintenance. Further, cultural norms may affect the consequences of different IER strategies. For example, previous research has shown that the use of intrapersonal expressive suppression is less detrimental to individuals in collectivist cultures versus more individualistic cultures [84, 85]. Future study should take these factors into account when evaluating the purpose and utility of different IER strategies.

### Clinical implications

Ultimately, additional understanding of IER can have a significant impact on clinical interventions. There are many unanswered questions about the most effective ways for people to support the affected person in managing emotions with the goal of ED recovery, such as which strategies must be disrupted to reduce ED behaviors and how the supportive person can best intervene when problematic IER is occurring. This is of particular relevance to treatments such as, but not limited to, family-based therapy [46], Maudsley Model of Anorexia Nervosa Treatment for Adults (MANTRA) [86], and Uniting Couples in the treatment of Anorexia Nervosa [87–89], which utilize the relationship between the recovering individual and their caregivers, family members, or other supports to facilitate recovery. In addition, treatments utilizing the cognitive interpersonal model of anorexia nervosa have shown success targeting problematic caregiver behaviors, such as accommodation [90]. Although these treatments leverage IER processes to achieve symptom improvements, for instance by reducing accommodation or using distraction to facilitate re-feeding, they have not been systematically evaluated using this framework. Furthermore, other eating disorders treatments incorporate aspects of emotion regulation and/or interpersonal relationships, such as dialectical behavior therapy (e.g., distress tolerance, emotion regulation, and interpersonal effectiveness modules) [45] and interpersonal psychotherapy for eating disorders [91], but do not specifically look at the interactions between these constructs. Consequently, the theoretical model of IER could help treatment developers perform process and dismantling studies assessing if these treatments are correctly identifying and modifying problematic IER behaviors that may be maintaining illness. Further, this theoretical model will inform the development and/or refinement of new treatments or modules to reduce problematic IER behaviors and ruptures in relationships.

This is important as the quality of interpersonal interactions may affect outcomes for people with EDs. In one qualitative study, women recovered from AN emphasized the importance of positive support and a non-judgmental attitude from health providers and support systems [92]. Further, given that therapy relationships are typically dyadic and interactional, increased understanding of IER processes and how they impact emotional and behavioral responses could be helpful in determining helpful therapist behaviors. For the individual, a focus on IER processes may help them to more effectively utilize their support systems, thereby enhancing effective ER and reducing interpersonal difficulties.

## Conclusion

Investigation of IER in EDs may yield insights into the ways in which interpersonal relationships influence how emotions are managed by individuals with EDs, and how different strategies perpetuate ED symptoms. IER represents a novel interdisciplinary field that can elucidate the unique role that other people play in guiding people with EDs through emotional distress. Ultimately, IER research holds promise to guide treatment recommendations, reduce distress and interpersonal problems in those with EDs, and empower support members to more effectively assist individuals with an ED through distress.

## Data Availability

Not applicable.

## Abbreviations

EDs: Eating disorders
ER: Emotion regulation
IER: Interpersonal emotion regulation

## Footnotes

1 In this review, we describe several interpersonal problems commonly endorsed by people with EDs to illustrate the importance of understanding interpersonal problems with this population; however, we note that this is not an exhaustive review of interpersonal problems in EDs.
